# Left ventricular non-compaction in a patient with ankylosing

**DOI:** 10.15171/jcvtr.2016.37

**Published:** 2016-12-30

**Authors:** Mehrnoush Toufan, Leili Pourafkari, Nader D. Nader

**Affiliations:** ^1^Cardiovascular Research Center, Tabriz University of Medical Sciences,Tabriz, Iran; ^2^University at Buffalo, Buffalo, New York, 14214, USA

**Keywords:** Ankylosing Spondylitis, Noncompaction Cardiomyopathy, Aortic Valve

## Abstract

A 58 years old male with a long-standing history of HLA-B27 positive ankylosing spondylitis presented with increasing fatigue and dyspnea on exertion. He had left ventricular dysfunction and enlargement, flail right coronary leaflet of aortic valve with severe eccentric aortic insufficiency along with left ventricular non-compaction in echocardiography. The most common cardiac manifestations of ankylosing spondylitis are aortic insufficiency and conduction disturbances. Involvement of myocardium, in the form of dilated cardiomyopathy and restrictive cardiomyopathy, has also been reported. This case presents a very rare association of ankylosing spondylitis with non-compaction cardiomyopathy.

## Introduction

Ankylosing spondylitis is categorized in the subgroup of seronegative spondylo-arthropathies and manifests with chronic inflammatory involvement of axial skeleton principally sacroiliac and spinal facet joints. More than half of patients also have peripheral joint involvement or enthesitis.^1^ Extra-articular manifestations including ophthalmic, pulmonary, neurologic, renal and cardiac involvement also occur with varying severity and contribute to the morbidity and morbidity.^2^ Cardiac involvement is reported in 2%-10% of the patients and is more common in long-standing disease with peripheral joint involvement.^3^ Diastolic dysfunction and involvement of myocardium, in the form of dilated cardiomyopathy and restrictive cardiomyopathy have also been reported.^4^ Hereby we report a rare association of ankylosing spondylitis with left ventricular non-compaction cardiomyopathy in a patient with long-standing disease.

## Case presentation

**Figure 1:**
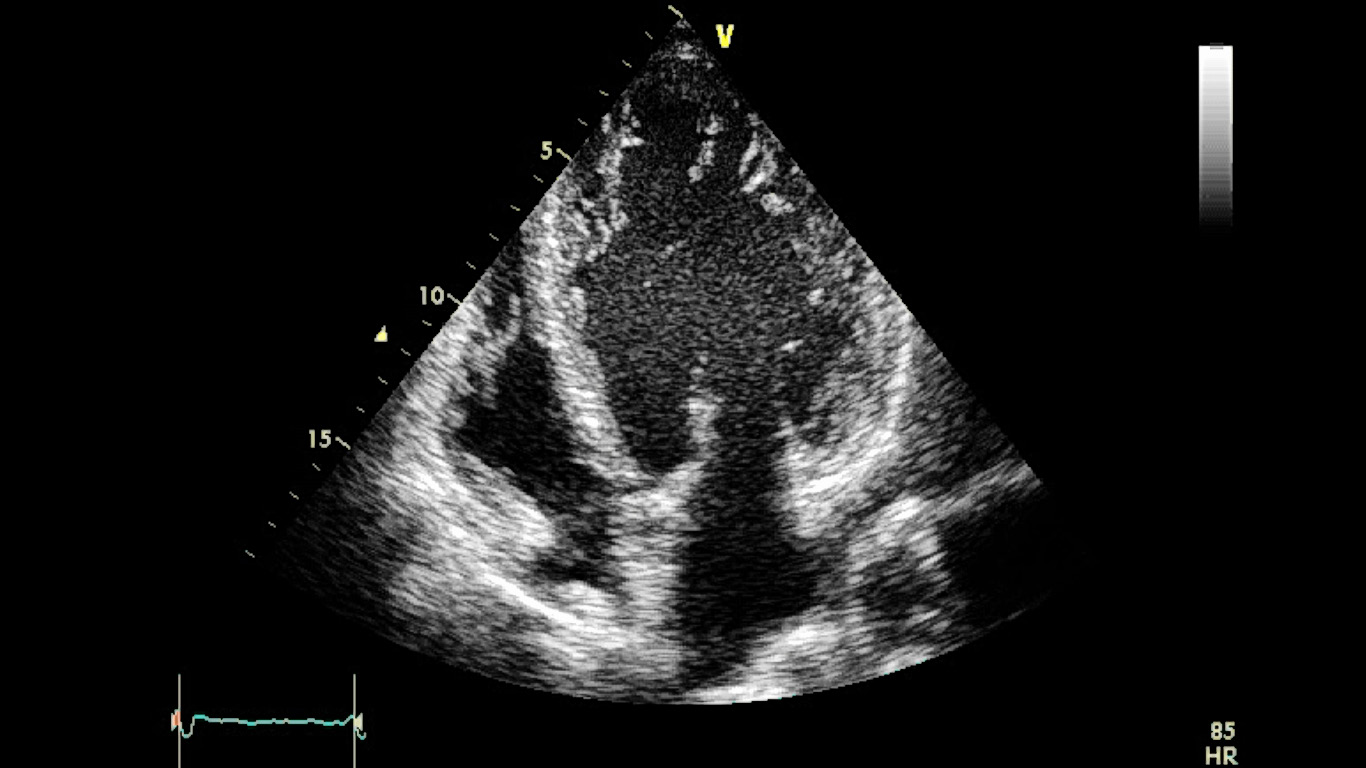
Four-chamber transthoracic echocardiogram showing non-compaction of both ventricles.

A 58 years old male with a 16-year history of HLA-B27^+^ ankylosing spondylitis presented with increasing dyspnea on moderate exertion and easy sense of fatigue. His joint pain was controlled effectively with non-steroidal anti-inflammatory drugs. He never smoked and the remaining of his medical history was unremarkable. Family history for cardiac or rheumatologic disorders was negative. On physical exam, he appeared tachycardic with a blood pressure of 135/60 mm Hg. Apical impulse was displaced to left and there was a diastolic murmur and S3 gallop on cardiac auscultation. In transthoracic echocardiography there was left ventricular enlargement with left ventricular end diastolic diameter of 7.2 cm and left ventricular end systolic diameter of 6.1 cm. Left ventricular ejection fraction was 32% (Supplementary Video 1). Right coronary cusp (RCC) of aortic valve (AV) was thick and fibrotic. RCC was flail causing significant defect in coaptation that yielded severe eccentric turbulent jet towards anterior leaflet of mitral valve (Supplementary Video 2). There was also biventricular non-compaction (Figure 1). Descending aorta holodiastolic flow reversal was also noted. In transesophageal echocardiography dilation of sinus of valsalva was noted (Supplementary Video 3). The patient refused surgery and opted to be managed medically.

## Discussion

Ankylosing spondylitis is a chronic inflammatory disease of the axial skeleton at large that predominantly affects young men.^5^ Cardiac involvement has long been recognized as an extra-articular manifestation of the disease and presents most commonly with aortic insufficiency and conduction abnormalities.^5^ Involvement of myocardium also reported, yet is not frequently encountered and remains less well recognized.^6,7^ Recently presence of left ventricular asynchrony has been shown in patients with ankylosing spondylitis.^6^ Cardiac involvement occurs more frequently in long-standing disease however they can precede skeletal symptoms.^7^ The prevalence of dilated cardiomyopathy in patients with ankylosing spondylitis is under-reported because it symptoms such as dyspnea on exertion may be attributed to the underlying disease. Restrictive cardiomyopathy secondary to amyloidosis has also been rarely been reported in patients with ankylosing spondylitis. Inflammatory processes and ensuing fibrosis have been implicated in the valvular dysfunction and conduction disturbances that associate with ankylosing spondylitis.^8^ In a recent study on patients with ankylosing spondylitis and no apparent cardiac involvement, increased aortic stiffness and decreased global myocardial performance have been reported which signifies the subclinical cardiac involvement in this group of patients.^3^

Left ventricular non-compaction may arise from the arrest of trabecular remodeling that happens during early fetal life. It is an uncommon entity that may present in isolated form or in association with other cardiac or systemic conditions. Though it is commonly considered a congenital disorder, the acquired cases have also reported. Right ventricle is involved in less than 50% of cases. Three major clinical manifestations are heart failure, arrhythmias, and systemic embolic events.^9^ It is speculated that left ventricular non-compaction may happen as an adaptive mechanism to certain hemodynamic conditions. Chronic left ventricular volume overload may be a potential trigger for increased trabeculation of myocardium. It is unclear whether left ventricular non-compaction cardiomyopathy has occurred secondary to aortic insufficiency or independently in this patient. The association of non-compaction cardiomyopathy with ankylosing spondylitis remains exceedingly rare.

## Ethical issues

The local ethics committee approved the study.

## Competing interests

None.

## Supplementary materials

videos 1

videos 2

videos 3 Supplementary file consists of videos 1, 2, and 3.
